# Effect of Diamond Surface Pretreatment and Content on the Microstructure and Mechanical and Oxidation Behaviour of NiAl/Fe-Based Alloys

**DOI:** 10.1155/2020/5149734

**Published:** 2020-08-07

**Authors:** Yaping Bai, Jianping Li, Jiajia Luo, Yongchun Guo

**Affiliations:** School of Materials and Chemical Engineering, Xi'an Technological University, Xi'an, 710021 Shaanxi Province, China

## Abstract

The effect of diamond surface pretreatment and content on the microstructure and mechanical properties of NiAl/Fe–*x* diamond (*x* = 0, 5, 10, 15, and 20 wt.%) alloys was investigated after mechanical alloying with subsequent hot-pressing sintering. The results showed that after the surface pretreatment, a complete transition layer containing W existed on the outer surface of the diamond grains, which improved the interfacial bonding strength of the diamond grains and NiAl/Fe matrix to an excellent level. As the diamond content increased, the compressive strength of the NiAl/Fe-based alloys declined, but the alloy with 10 wt.% diamond had a higher value than that of the other NiAl/Fe-based alloys. Short cracks and transgranular fracture were observed in the fracture surface of all materials. For the material with 20 wt.% diamond, intergranular fracture was obvious, and many diamond particles appeared along the fracture direction, which caused the compressive strength to be the lowest of the samples considered in this study. After the addition of diamond, the oxidation resistance of NiAl/Fe-based alloys decreased due to a loose oxidation layer and diamond graphitization. The thermal conductivity of the alloy first increased and then decreased with increasing diamond content. A NiAl/Fe-based alloy with 15 wt.% diamond demonstrated the maximum thermal conductivity of 53.2 W/(m·k) at 600°C among the samples in this study.

## 1. Introduction

It is generally known that the cylinder head of a diesel engine often contacts high-temperature and high-pressure gas, and it therefore bears substantial heat and mechanical loads [1–4]. Materials in severe working conditions must possess not only high strength and creep resistance but also excellent thermal conductivity and corrosion resistance [5–9]. Cast iron (7.6 g/cm^3^), as the present cylinder head material, is not readily fulfilling the requirements of engines with increasing power. In particular, the mechanical properties of cast iron deteriorate sharply when the environment temperature increases to 400°C [10, 11]. Therefore, iron-based materials with low density, excellent mechanical properties, and thermal conductivity should be developed.

In 2015, scientists at the Pohang University of Science and Technology in South Korea found that during the annealing process, nickel can react with aluminium to produce nanoscale B2-ordered NiAl crystals. The B2 crystals have excellent shear resistance, so steel containing B2-ordered NiAl crystals can demonstrate high strength and high ductility [12]. Thus, the B2-ordered NiAl phase, as a particle reinforcement, is a good choice for improving the mechanical properties of iron-based alloys. In addition, it is known that the cost of synthetic diamond is low. It has not only high hardness and chemical stability but also excellent thermal conductivity (600-2000 W/m·K) and thermal stability, so it is now widely used for polishing, as a particle reinforcement and as a composite coating, for example [13–15]. The interface bonding strength between the matrix and diamond may be primarily due to mechanical mixing without a transition layer [16]. The interface between the Fe matrix and diamond particles is the major determinant of the Fe/diamond composite properties. Therefore, pretreatment of the diamond powders is very important for ensuring adhesion between the diamond and matrix. In recent years, research has focused on surface pretreatments of the diamond to solve such problems [16–18]. Scholars found that tungsten layers can be coated on diamond by a microvacuum evaporation diffusion method and then, diamond/copper composites can be prepared by vacuum pressureless infiltration. The resulting full uniform plating layer on the diamond/copper interface has high thermal conductivity [16].

Therefore, B2-ordered NiAl/Fe-based alloys with different contents of diamond powders before/after pickling treatment are prepared by mechanical alloying with subsequent hot-pressing sintering based on previous research in our laboratory and are discussed herein. Then, the microstructure, mechanical properties, oxidation resistance, and thermal conductivity at RT ~600°C are studied. The study results can provide theoretical and technical support for future cylinder head materials.

## 2. Experimental Procedures

### 2.1. Material Preparation

The B2-ordered NiAl powders were prepared by the following ball milling process. The Ni powder and Al powder with a 1 : 1 atomic ratio were homogenized by ball milling under an argon gas protective atmosphere by a Pulverisette P5-type (Germany) variable-frequency planetary ball mill. The test parameters were as follows: the ball material ratio was 10 : 1, the speed was 250 r/min, and the process was on for 30 min and then stopped for 30 min for a total of 70 h.

Diamond-/NiAl-reinforced iron-based alloys were prepared by hot-pressing sintering after the surface pretreatment of the diamond powders. The diamond exhibited excellent properties, such as high hardness, low density, and high thermal conductivity, but it had poor interface bonding with the Fe-based alloys. Therefore, surface pretreatment of the diamond powders must be carried out first. The surface pretreatment of the diamond powders was as follows. The diamond powder, Cu powder, and W powder were mixed according to a mass ratio of 75% : 20% : 5%. Due to the good thermal conductivity of copper powder, the heat in the alloy powder can be evenly diffused without heat accumulation at high temperature. Then, the powders were treated as follows. They were pickled in acid (in 10 wt.% hydrochloric acid at 60°C for 30 min) → cleaned with acetone for 20 min → cleaned with alcohol for 20 min → cleaned with acetone for 20 min → dried. Then, the mixed powders were vacuum heat treated at 1000°C for 60 min. The redundant tungsten powders were removed by sieving the powder in 300-mesh, 400-mesh, and then 600-mesh sieves successively. Finally, the tungsten-coated diamond powder was obtained.

The NiAl powders and iron powders with 0 wt.%, 5 wt.%, 10 wt.%, 15 wt.%, and 20 wt.% diamond powder were mixed with a YXQM-type planetary mill at a ball : material ratio of 3 : 1 at a speed of 100 r/min for 5 h.

Thirty grams of the milled powders was packed in a graphite mould (inner size Ø30 mm × 50 mm) under a continuous pressure of 20 MPa using a hot-pressing sintering apparatus (ZT-40-20Y, Shanghai Chen Hua Electric Furnace Co., Ltd., China) that operated under a vacuum of 7 × 10^−2^ Pa, heated at a rate of 10°C/min, and then consolidated the material at 1050°C for 60 min.

### 2.2. Microstructure and Performance Test

The densities were measured by the Archimedes method. The phase analysis was evaluated by X-ray diffraction (XRD) using an X-ray diffractometer (Rigaku D/Max-RB) with Cu K*α* radiation (wavelength = 0.15418 nm) at 40 kV and 100 mA. The microstructure and interface bonding were characterized by scanning electron microscopy (SEM) on an instrument equipped with an energy dispersive spectroscopy (EDS) and also transmission electron microscopy (TEM). Samples with a size of 5 mm × 5 mm were cut from the alloys by an electrical discharge wire-cutting machine for hardness testing. The bulk hardness was measured on an HRS-150 Digital Rockwell Hardness Machine with an applied load of 1470 N. Specimens with a size of 4 mm × 4 mm × 7 mm were cut from the sintered bulk samples by electrodischarge machining for compression tests. Before the compression tests, the six surfaces of the specimens were polished first. The room temperature compression performance was tested with an electronic universal tensile test machine (D2-0200-1) with a strain rate of 5 × 10^−3^/s. The oxidation behaviour of the composites was tested on a HENVEN-HJ integrated thermal analyser at 600°C for 25 h and 250 h. The nominal sample dimension was 20 mm × 20 mm × 1.5 mm, and the introduced air flowed at 50 ml per minute during the oxidation experiment. Before the oxidation test, the specimens were predried to a constant weight. The thermal conductivity of the alloy with *Φ*12.5 × 2.5 mm was measured by a NETZSCH-LFA457 laser thermal conductor at room temperature, 100°C, 200°C, 300°C, 400°C, 500°C, and 600°C.

## 3. Results and Discussion

### 3.1. Phase Composition and Microstructure

**Figure 1:**
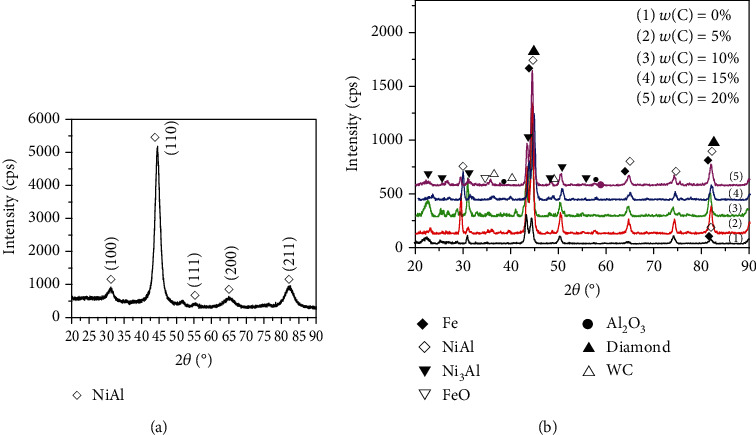
XRD patterns of NiAl powders and NiAl/Fe-based alloys with different diamond contents: (a) as-milled NiAl powders; (b) sintered NiAl/Fe-based alloys.

The XRD diffraction patterns of the as-milled NiAl powder and NiAl/Fe-based alloys with different diamond contents after the sintering process are presented in Figures 1(a) and 1(b), respectively. From Figure 1(a), it can be observed that the diffraction peaks from the (100), (110), and (211) planes are present in the XRD pattern of the NiAl powders, especially the (110) diffraction peak, which is the strongest. It can be concluded from the XRD data that NiAl powder with a B2 structure is successfully obtained.  Figure 1(b) indicates that superlattice reflection peaks of NiAl are still present in all the sintered samples. In addition, a small amount of B2-ordered NiAl phase transforms to Ni_3_Al at the high temperature during the sintering process, so the Ni_3_Al phase diffraction peak also appears in the XRD pattern of the bulk sample. The WC diffraction peaks obviously increase with increasing diamond content, which infers that a WC coating layer is formed on the diamond surface. In addition, during the sintering process under a low-vacuum environment, some Fe and Al elements are oxidized to FeO and Al_2_O_3_, respectively.

**Figure 2:**
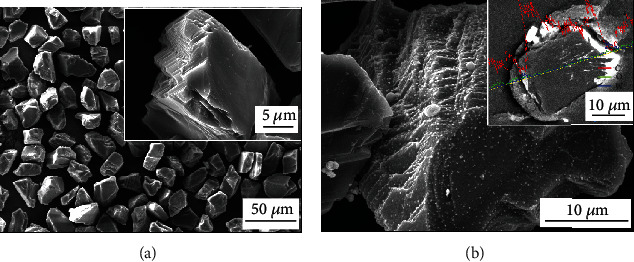
Micromorphology and line scanning results of diamond before and after pretreatment: (a) before pretreatment; (b) after pretreatment.

Figure 2 shows the micromorphology and line scanning results of the diamond before and after the surface pretreatment. After the pretreatment, the diamond surface becomes rough, and the results of the line scanning analysis show that there is a WC layer on the surface, which is in accordance with the results of the XRD test shown in Figure 1(b). The increased surface roughness and WC formation are beneficial to the bonding with the matrix phase during the sintering process.

**Figure 3:**
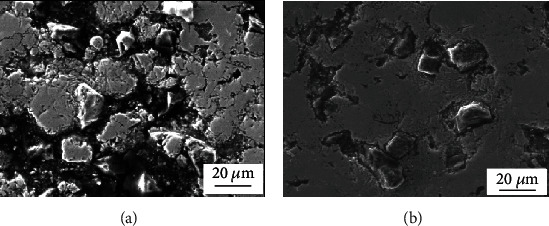
SEM images of NiAl/Fe-based alloys with 20 wt.% diamond: (a) before pretreatment; (b) after pretreatment.

Figure 3 shows SEM images (secondary electron images) of NiAl/Fe-based alloys with 20 wt.% diamond before and after pretreatment. From the secondary electron images (SE) of NiAl/Fe-based alloys with untreated/pretreated 20 wt.% diamond, there is an obvious gap between the untreated diamond and the NiAl/Fe matrix material shown in Figure 3(a); however, the interface between the pretreated diamond powder and the matrix is very close shown as the Figure 3(b), which indicates that the bonding strength of the pretreated diamond and the matrix is improved. All these indicate that the addition of tungsten can prevent the graphitization of diamond at high temperature and can improve the bonding strength between diamond and alloy matrix.

**Figure 4:**
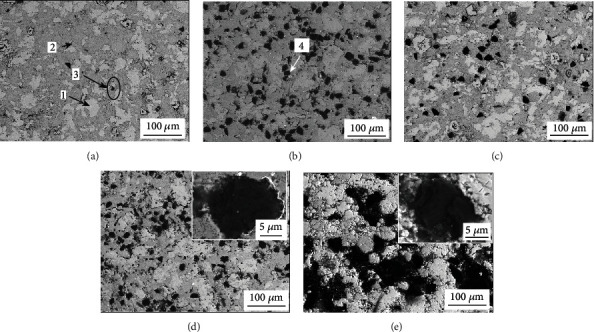
SEM images of NiAl/Fe-based alloys with different diamond contents: (a) 0 wt.%; (b) 5 wt.%; (c) 10 wt.%; (d) 15 wt.%; (e) 20 wt.%.

**Figure 5:**
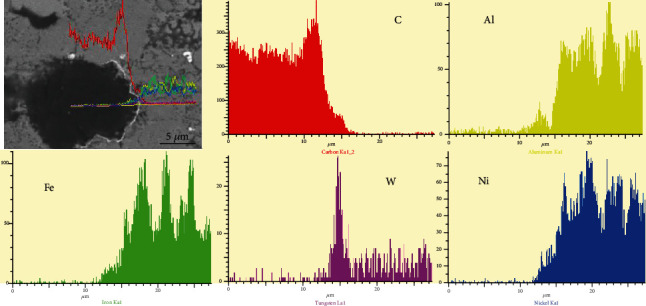
Line scanning results of NiAl/Fe-based alloys with 15 wt.% diamond shown in Figure 4(d).

Figure 4 shows SEM images (backscattered electron images) of the NiAl/Fe-based alloys with 0 wt.%, 5 wt.%, 10 wt.%, 15 wt.%, and 20 wt.% diamond. The corresponding point EDS analysis is shown in Table 1. The line scan result in Figure 4(d) is shown in Figure 5. Combined with Figure 4(a) and the EDS results, it can be concluded that the white areas (point 1) are iron matrix, the grey areas (point 2) are mainly NiAl phase, and the black particles (point 3) are FeO phase with a grain size of approximately 10 *μ*m. With the addition of diamond particles, homogeneous granular black particles appear in the microstructure, but when the diamond content increased to 20%, the diamond forms a substantial amount of agglomerates. From Figure 5, a relatively complete WC transition layer can be seen between the diamond and matrix. These data are enough to prove that after the diamond surface pretreatment, a complete WC layer forms on its outer surface. All the analyses are consistent with the XRD results shown in Figure 1.

**Figure 6:**
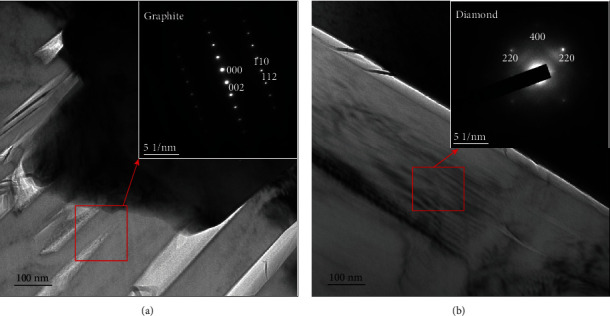
Transmission electron micrographs and selected area electron diffraction patterns of NiAl/Fe-based alloys with 10 wt.% diamond: (a) graphite structure; (b) diamond structure.

The bonded area between the diamond and matrix was further magnified by transmission electron microscopy (TEM), as shown in the micrograph of the NiAl/Fe alloy with 10 wt.% diamond in Figure 6. In Figures 6(a) and 6(b), it can be seen that the diamond and matrix forms a good interface bond with no obvious gap. Further analysis proves that there are two forms of C present: a strip-like grey-white phase in Figure 6(a) and pure grey plate-like phase in Figure 6(b). The calibration results of the diffraction patterns indicate that the grey-white strip-like material is a graphite phase and that the pure grey plate-like material is a diamond phase. All these results indicate that diamond graphitization occurs during hot-pressing sintering, which also confirms that the black worm-like substance in the scanning photograph of Figure 4(d)is graphite.

### 3.2. Mechanical and Thermal Physical Properties

**Figure 7:**
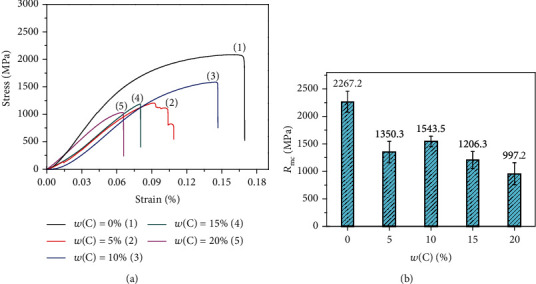
Compressive stress-strain curve and compressive strength of NiAl/Fe-based alloys with different diamond contents: (a) stress-strain curve; (b) compressive strength.

**Figure 8:**
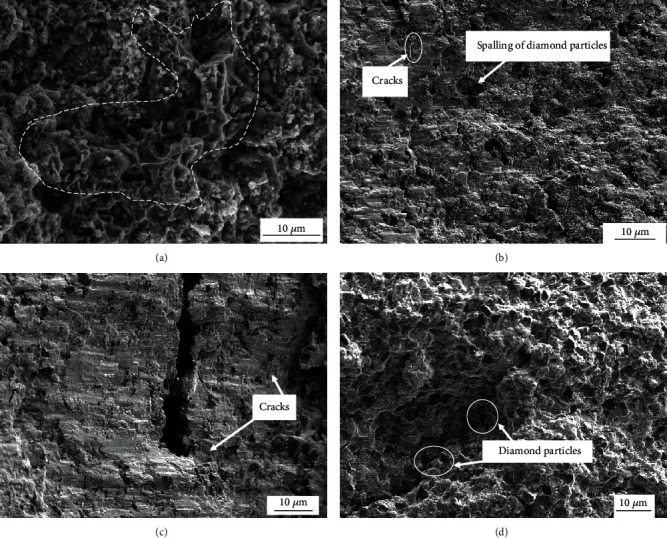
Fracture surfaces of NiAl/Fe-based alloys with different diamond contents: (a) 0 wt.% (b) 5 wt.%; (c) 10 wt.%; (d) 20 wt.%.

Figure 7 shows the compressive stress-strain curve of the NiAl/Fe-based alloys with different diamond contents at room temperature.  Figure 8 shows the fracture surfaces of the NiAl/Fe-based alloys with different diamond contents. From Figure 7, it can be seen that the compression ratio of the NiAl/Fe-based alloys is larger than that of the NiAl/Fe-based alloys with the diamond addition at the same temperature. These results are due to the second phase addition acting as a crack source, as shown in Figure 8(b). From Figure 8(d), it is clear that cracks extend along the diamond boundary, and intergranular fracture is obvious, and many diamond particles appear along the crack. However, the fracture is nearly smooth without obvious diamond particle spalling, as shown in Figure 8(c). Therefore, NiAl/Fe-based alloys with 10 wt.% diamond have high compressive strength and excellent compression deformation.

**Figure 9:**
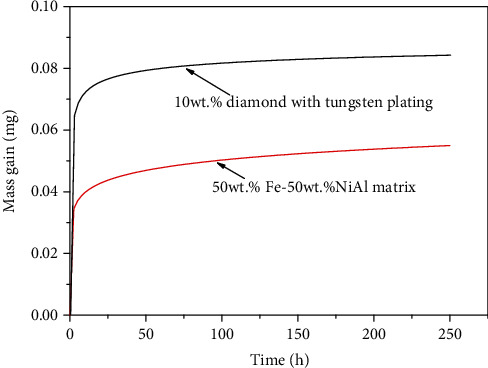
Oxidation weight gain-oxidation time curve of NiAl/Fe-based alloys with 0 wt.% and 10 wt.% diamond.

Figure 9 shows the oxidation weight gain-oxidation time curve of NiAl/Fe-based alloys with 0 wt.% and 10 wt.% diamond after treatment at 600°C for 250 h. The oxidative weight gain of the NiAl/Fe-based alloys with 10 wt.% diamond becomes steady during the last oxidation stage (after 25 h). It can also be seen that the oxidation weight gain of NiAl/Fe-based alloys increases after 10 wt.% diamond addition. This is because graphitization of the diamond surface occurs during the oxidation test. According to the research results by Guo et al., both the shell of the iron atoms and the electrons of the diamond atoms can attract diamond atoms and gradually form graphite structures; therefore, it is concluded that iron accelerates the process of diamond graphitization [19]. This explanation is consistent with the XRD results shown in Figure 1.

**Figure 10:**
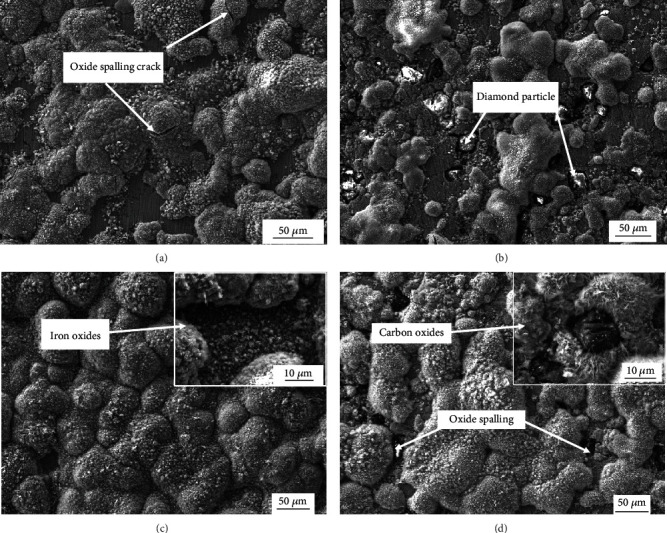
SEM images of NiAl/Fe-based alloy surface morphologies after oxidation for 25 h and 250 h: (a) NiAl/Fe-based alloys with 0 wt.% diamond—25 h; (b) NiAl/Fe-based alloys with 10 wt.% diamond—25 h; (c) NiAl/Fe-based alloys with 0 wt.% diamond—250 h; (d) NiAl/Fe-based alloys with 10 wt.% diamond—250 h.

Figure 10 shows SEM images of NiAl/Fe-based alloy surface morphologies after oxidation for 25 h and 250 h. From Figure 10(a), it is clear that the NiAl/Fe alloy surface oxide layer is basically formed after oxidation for 25 h, and cracks from oxidation spalling occurred in the area where the oxide layer is thick. During oxidation for 250 h, the nonoxidized areas are continuously oxidized, and the thicker areas continuously peel off, so a uniform oxide film on the sample surface is formed. The oxidation areas on the surface of the NiAl/Fe alloy are reduced with a 10 wt.% diamond addition, and a thick oxidation film around the diamond particles cannot be seen (as shown in Figure 10(b)). Obviously, the diamond addition reduces the oxidation degree of the alloy surface. From Figure 10(d), it can be seen that oxide spalling is present on the surface of the NiAl/Fe-based alloy with 10 wt.% diamond after the 250 h oxidation test. Compared with that of the NiAl/Fe-based alloy shown in Figure 10(c), the surface oxide layer of the NiAl/Fe-based alloy with 10 wt.% diamond is clearly looser.

**Figure 11:**
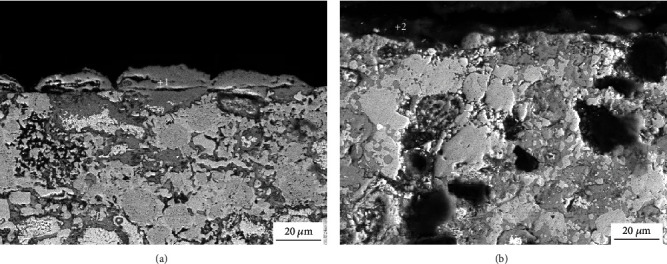
Cross-section image of the oxide film of the NiAl/Fe-based alloy after oxidation for 25 h: (a) NiAl/Fe-based alloys with 0 wt.% diamond; (b) NiAl/Fe-based alloys with 10 wt.% diamond.

Figure 11 shows the SEM image of the cross section of the iron-based alloys after the 25 h oxidation test. The oxidation of the NiAl/Fe-based alloys with 0 wt.% diamond mainly occurs on the sample surface, while the internal oxidation of the sample is slight. When 10% diamond is added, both the sample surface and interior parts have different oxidation degrees, but the internal oxidation of the sample is particularly substantial. EDS analysis of the surface oxidation region was completed, and the results are shown in Table 2. The oxide film contains Fe, Ni, Al, and O when diamond addition is 0 wt.%. However, after adding 10 wt.% diamond, the Al content in the oxide layer is only 0.52%, the Ni element completely disappears, and the oxide film is almost entirely Fe oxides. The Ni-based oxides and Al-based oxides on the surface of NiAl/Fe-based alloys with 0 wt.% diamond can densify the oxide film on the surface of the alloy, which can hinder further oxidation. However, the oxidation of Al and Ni in the alloy is reduced or even eliminated after diamond is added. In addition, diamond also destroys the integrity of the oxide layer on the alloy surface. All these factors increase the oxidation inside the alloy.

**Figure 12:**
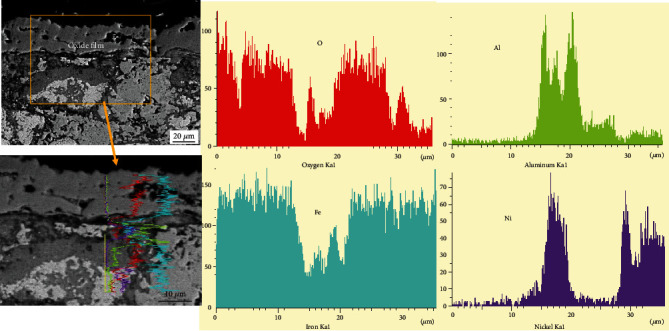
Element distribution in the cross section of the oxide film of the NiAl/Fe-based alloy with 0 wt.% diamond after oxidation for 250 h.

**Figure 13:**
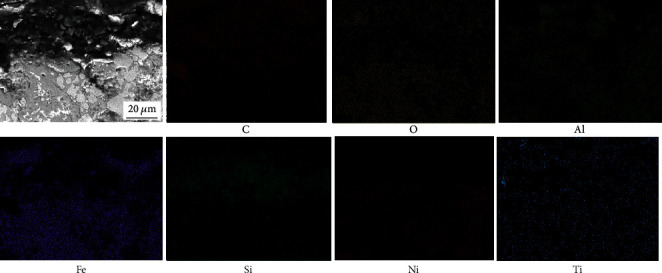
Element distribution in the cross section of the oxide film of the NiAl/Fe-based alloy with 10 wt.% diamond after oxidation for 250 h.

Figures 12 and 13 show the element distribution of the cross section of the oxide film of the NiAl/Fe-based alloy with 0 wt.% diamond after oxidation for 250 h.  Figure 12 shows that the oxides are mainly iron oxides. Further observation indicates that there is a small amount of alumina and iron oxides exist in alloy matrix. For the oxide film on the NiAl/Fe-based alloy with 10 wt.% diamond, the surface oxide layer is somewhat loose. It can be inferred from the element distribution in the cross section of the oxide film shown in Figure 13 that a substantial amount of Si is distributed in the oxide film because silicone phenolic plastics are the metallographic mosaic powder. It also shows a minor amount of iron oxides, and aluminium oxides appear in the surface layer. These results indicate that the oxide layer is relatively loose. This can explain why the NiAl/Fe-based alloy with 10 wt.% diamond has a higher oxidation weight gain.

**Figure 14:**
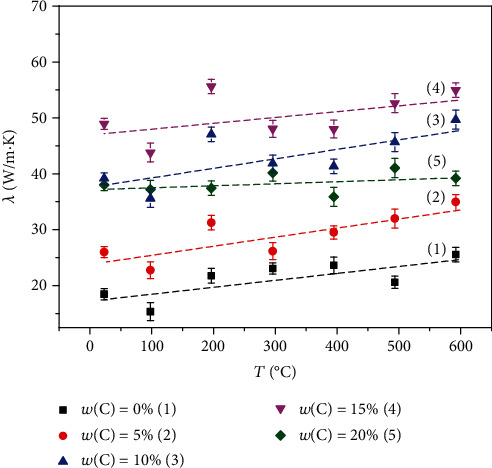
Thermal conductivity of NiAl/Fe alloy at different temperatures with diamond addition.

The thermal conductivity of the NiAl/Fe alloy with different diamond contents with increasing temperature is shown in Figure 14. The thermal conductivity of the material increases substantially after diamond is added. As the diamond amount increases, the thermal conductivity of the material first increases and then decreases. When the content of diamond is 15 wt.%, the thermal conductivity of the material reaches its peak at each temperature, and the thermal conductivity is 53.2 W/(m·K) at 600°C. It is generally known that diamond is a natural material with the highest thermal conductivity among known materials (600-2200 W/m·K). Adding diamond to the matrix material can effectively improve the overall thermal conductivity of the material. However, with an increasing amount of diamond, the number of interface and sintering voids increases, so the electronic movement between crystals is hindered and the thermal conductivity of the material is reduced.

The addition of surface-pretreated diamond to NiAl/Fe-based alloys reduces the density and improves the thermal conductivity, but it slightly degrades the mechanical properties. Therefore, the densification of the material is further improved by hot isostatic pressing (HIP), and the overall mechanical properties and oxidation resistance of the material are further improved. Thus, low-density iron-based alloys with excellent mechanical properties, oxidation revsistance, and thermophysical properties can be obtained with this approach.

## 4. Conclusions

NiAl/Fe-based alloys with 0, 5, 10, 15, and 20 wt.% diamond-reinforced particles were prepared by hot-pressing sintering after the surface pretreatment of the diamond powders. From the microstructure, mechanical properties, oxidation resistance, and thermal conductivity analysis, the following conclusions can be summarized:

1. A complete transition layer containing W existing outside diamond was gained after surface pretreatment of the diamond powders, which improved the interface bonding of the diamond grains and NiAl/Fe matrix to an acceptable level
2. The compressive strength of NiAl/Fe-based alloys decreased after adding the pretreated diamond, but as the diamond addition increased, the alloy with 10 wt.% diamond obtained a higher compressive strength than that of other diamond contents
3. After diamond addition, the corresponding oxidation resistance decreased because of the loose oxide layer and diamond graphitization of the NiAl/Fe-based alloys
4. The thermal conductivity of the alloy increased first and then decreased as the diamond content increased. When the diamond content was 15 wt.%, the thermal conductivity of the alloy reached the maximum value of 53.2 W/m·K at 600°C obtained in this study

## Figures and Tables

**Table 1:** EDS results of the points marked in Figures 4(a) and 4(b).

Points	Fe (wt.%)	Ni (wt.%)	Al (wt.%)	C (wt.%)	O (wt.%)
1	96.73	0.86	1.58	0.83	—
2	6.47	68.66	23.79	1.08	—
3	58.74	—	—	3.64	37.62
4	1.92	2.11	2.50	89.66	3.81

**Table 2:** Point analysis of the cross section of the oxide film of the NiAl/Fe-based alloy shown in Figure 11.

Points	Fe (wt.%)	Ni (wt.%)	Al (wt.%)	O (wt.%)
1	23.87	4.14	9.57	64.42
2	33.55	—	0.52	65.93

## Data Availability

The data used to support the findings of this study are included within the article.
